# Human umbilical cord plasma derived exosome changed the miRNAs expression and inhibits inflammation response in traumatic spinal cord Injury

**DOI:** 10.1016/j.ibneur.2026.01.002

**Published:** 2026-01-05

**Authors:** Shima Jahanbaz, Hamid Reza Mosleh, Shahram Darabi, Hadise Taheri, Hooman Kazemi Mirni, Maryam Bahrami, Abbas Aliaghaei, Amin Karamian, Reza Bahar, Maral Hasanzadeh, Foozhan Tahmasebinia, Amirreza Beirami, Hojjat-Allah Abbaszadeh, Leila Darabi

**Affiliations:** a Department of Biology and Anatomical Sciences, School of Medicine, Shahid Beheshti University of Medical Sciences, Tehran, Iran; b Laser Application in Medical Sciences Research Center, Shahid Beheshti University of Medical Sciences, Tehran, Iran; c Proteomics Research Center, Faculty of Paramedical Sciences, Shahid Beheshti University of Medical Sciences, Tehran, Iran; d Rayan stem cells and Regenerative medicine research center, Ravan Sazeh Company, Tehran, Iran; e Department of Neurology, Tehran Medical Science Branch, Amir Al-Momenin Hospital, Islamic Azad University, Tehran, Iran

**Keywords:** Functional recovery, Exosome, Inflammation, MiRNAs, Spinal cord injury

## Abstract

Spinal cord injury (SCI) is a debilitating neurological condition that leads to physical dependence, substantial financial burden, and psychological stress. Current for SCI, such as stem cell therapy, pharmacological interventions, and neural implants offer limited functional recovery. Among emerging strategies, exosome-based therapies in nerve damage can reduce neuroinflammation and promote neural repair by angiogenesis and neurogenesis. MicroRNAs (miRNAs) are key modulators of inflammatory and regenerative pathways in SCI. Specifically, miR-19a-3p, miR-19b-3p, and miR-27b have been implicated in regulating neuroinflammatory responses, neuronal survival, and tissue remodeling. Dysregulation of these miRNAs following SCI can exacerbate inflammation and hinder recovery. In this study, exosomes were extracted and characterized using flowcytometry for surface markers CD81 and CD9, scanning electron microscopy (SEM), dynamic light scattering (DLS), and Zeta potential analysis. Thirty-two female rats were randomly assigned into four groups: laminectomy only, contusion, contusion + PBS, and contusion + exosomes. SCI were induced using contusion model and thirty minutes after the injury, the exosome-treated group received an intravenous injection of 100 μl of exosomes via the tail vein for 7 days. Motor and behavioral functions were assessed through the open-field test, Basso, Beattie, and Bresnahan (BBB) scale and narrow beam test (NBT). Eight weeks after the SCI, real time PCR, Western blotting was utilized to assess changes in inflammatory cytokines, while histological changes were observed using hematoxylin and eosin (H&E) staining and stereology. In vivo experiments showed that the administration of exosomes significantly enhanced functional recovery and behavioral test outcomes following SCI. The treatment also resulted in a significant reduction in inflammatory cytokine levels and a marked decrease in the size of the cavity in the group treated with exosomes. Molecular analysis revealed that exosome therapy modulated the expression of miR-19a-3p, miR-19b-3p, and miR-27b, which are key regulators of neuroinflammation and neural repair. These findings suggest that exosomes hold strong therapeutic potential for treating SCI by modulating inflammation and promoting neural repair. Collectively, these findings indicate a potential mechanism through which exosomes exert their neuroprotective effects, particularly by regulating inflammatory and regenerative pathways.

## 1 Introduction

Spinal cord injury (SCI) poses a significant clinical challenge characterized by profound neurological deficits and limited regenerative capacity (Abbaszadeh et al., 2018, Tian et al., 2023, Vafaei-Nezhad et al., 2020, Vafaei-Nezhad et al., 2019, Venkatesh et al., 2021). Despite intensive research efforts, effective treatments to mitigate SCI-induced damage and promote functional recovery remain elusive (Ramer et al., 2014). In recent years, exosome-based therapy has emerged as a promising approach for SCI management, offering potential advantages over traditional therapeutic modalities (Liu et al., 2021, Yi and Wang, 2021). Exosomes, which are nanosized extracellular vesicles released by cells, are enriched with bioactive molecules capable of modulating cellular processes and promoting tissue repair (Bjorge et al., 2018, Desideri et al., 2021). Among exosome sources, human umbilical cord blood (hUCB) has garnered attention due to its abundance, accessibility, and potent regenerative properties (Feng et al., 2021). At the molecular level, SCI initiates a series of pathological processes, such as inflammation, oxidative stress, and glial activation, exacerbating tissue damage and impeding recovery (Anjum et al., 2020, Fakhri et al., 2022). Following SCI, pro-inflammatory cytokines like interleukin-6 (IL-6) and interleukin-1β (IL-1β), are prominently upregulated following SCI, contributing to neuroinflammation and secondary injury progression (Garcia et al., 2016, Hellenbrand et al., 2024). Concurrently, reactive oxygen species (ROS) production increases, overwhelming endogenous antioxidant defenses and exacerbating tissue damage (He et al., 2023). Glial fibrillary acidic protein (GFAP), an indicator of astrocyte activation, is robustly upregulated following SCI, contributing to scar formation and inhibiting axonal regeneration (Abdelhak et al., 2022, Brenner, 2014). Emerging evidence suggests that microRNAs (miRNAs) play a pivotal role in regulating these pathological mechanisms in SCI (C. Zhang et al., 2023). Specifically, miR-19a-3p, miR-19b-3p, and miR-27b have been implicated in the modulation of neuroinflammation, neuronal survival, and tissue remodeling (Acharya et al., 2020, Silvestro and Mazzon, 2022, Tregub et al., 2023). Dysregulation of these miRNAs after SCI has been linked to excessive inflammation, impaired neuroprotection, and hindered repair processes. Exosome-based therapies, particularly those derived from hUCB, may exert their therapeutic effects by delivering miRNAs that restore molecular homeostasis, suppress inflammatory signaling, and enhance neuroregeneration. Recent preclinical studies have highlighted the potential therapeutic benefits of stem cell-derived exosomes in attenuating SCI-induced molecular alterations and promoting neurodegeneration (Herbert et al., 2022, Schepici et al., 2023, Wang et al., 2018, Yari et al., 2022). These exosomes exhibit immunomodulatory properties, suppressing pro-inflammatory cytokine production and fostering an anti-inflammatory microenvironment conducive to tissue repair. Furthermore, stem cell-derived exosomes mitigate oxidative stress by reducing ROS level and enhancing cellular antioxidant defenses, thereby preserving tissue integrity and facilitating regeneration (Che et al., 2024; Z.-X. Zhang et al., 2023; W. Zhang et al., 2023, Zhao et al., 2022). Additionally, exosome therapy modulates glial activation, leading to reduce GFAP expression and promoting a permissive environment for axonal outgrowth (Che et al., 2024, Goncalves et al., 2015). The intricate interplay of inflammatory mediators, oxidative stress, miRNA regulation, and glial activation underscores the multifaceted nature of SCI pathology. By elucidating the molecular mechanisms underlying the therapeutic potential of hUCB-derived exosomes—particularly their ability to modulate miRNA expression — this study aims to provide critical insights into their neuroprotective and regenerative effects in SCI. Understanding these interactions may pave the way for innovative therapeutic strategies aimed at improving outcomes and quality of life for individuals affected by SCI.

## 2 Materials and methods

### 2.1 Animal

To meet the research goals, 32 adults female Wistar rats, with weights ranging from 250 to 300 g, were selected for this research study. The rats were sourced from Shahid Beheshti University - Laboratory Animal Center. All experimental procedures were conducted in accordance with the guidelines set by the Ethics Committee on Animal Experimentation of Shahid Beheshti University of Medical Sciences. The rats were housed individually in standard laboratory cages under controlled environmental conditions, which included a humidity level of 50–60 %, 12-hour light/dark cycle, and temperature range of 21–23°C. They had unrestricted access to water and food throughout the study.

### 2.2 Animal model of spinal cord injury

In this experiment, 32 Female rats were divided randomly into four groups: First, Group A underwent only a laminectomy; Group B underwenr spinal cord contusion; Group underwent C contusion followed by phosphate-buffered saline (PBS) treatment (contusion + PBS); and Group D underwent contusion followed by exosome treatment for one week (contusion + exosome). The rats were sedated with a combination of xylazine (10 mg/kg) and ketamine (80 mg/kg) before undergoing a laminectomy at the T8 vertebra. SCI was induced using the NYU weight-drop method, which involved dropping a 10 g weight from a height of 2.5 cm onto the exposed spinal cord. Following injury, the wound was closed by suturing muscles and fascia layers, and the skin incision was sutured in two layers. Post-surgery, the animals’ urinary bladders were manually expressed twice daily to facilitate recovery. To prevent infection, gentamicin (0.01 mg/kg) was administered through intraperitoneal injection for a duration of five days. Thirty minutes post-injury, Group D received 100 μl of exosomes resuspend in 1 mL of PBS via tail vein injection once daily for 1 week, while Group C received the same volume of PBS (1 mL) via tail vein injection 30 min after the injury at the same time point (Kwon et al., 2002).

### 2.3 Exosome extraction and conformation

A 50 mL sample of umbilical cord blood was collected immediately after delivery and stored at 4°C for 30 min. Subsequently, the sample was centrifuged at 1500 rcf for 15 min at the same temperature to isolate the plasma. The plasma was then stored at −80°C for future use. Exosome extraction from the plasma involved a three-step ultracentrifugation process at 10000 rcf for 20 min, 50000 rcf for 30 min, and 100000 rcf for 60 min), all conducted at 4°C. Surface markers CD81 and CD9 were identified using flowcytometry with phycoerythrin (PE)–conjugated antibodies. Particle size distribution and surface was measured and assessed through Zeta potential analysis and dynamic light scattering (DLS), respectively. The exosomes were resuspended in PBS and stored until use. For electron microscopy, the sample was prepared as a thin layer on a gel-coated scaffold placed on a coverslip. The coverslip was fixed with 2.5 % glutaraldehyde, followed by protein cross-linking to stabilize the sample, which was then coated with gold and examined under an scanning electron microscope (SEM). The same exosome batch was used for all in vivo experiments to ensure experimental consistency.

### 2.4 BBB (basso, beattie, bresnahan) test

Behavioral assessments were carried out weekly following the injury and continued until the study's conclusion at the end of the second month. These tests aimed to evaluate motor function recovery in the animals. The testing process was recorded on video, with the results analyzed using MGI Video Wave 5 software. To measure the animals' immobility, the baseline scores from the Basso, Beattie, and Bresnahan (BBB) behavioral assessment obtained after injury were compared to the post-treatment scores. The BBB scale rates locomotion quality ranging from 0 to 21, with 0 represents complete paralysis and 21 indicates normal locomotion.

### 2.5 Narrow beam test (NBT)

NBT was used to assess motor coordination and sensory function in the animal models. Initially, it was performed on all rats before any intervention to establish a baseline. The rats were trained to walk across a wooden beam, measuring 80 cm in length and 4 cm in width. Following the induction of the spinal cord injury, the test was administered weekly. Each rat's performance score was recorded after every testing session, and the collected data were analyzed at the study’s conclusion.

### 2.6 Open-field test

At the conclusion of the study, the locomotor activity of the animals was evaluated using the open-field test. In this procedure, the animals were placed in a 90 × 90 cm square arena's corner. A video tracking system automatically recorded the total distance traveled by the rats. To ensure precision, the arena was cleaned with 70% alcohol and allowed to dry before each trial, eliminating any urine or olfactory cues from previous tests. Additionally, all behavioral evaluations took place in a calm, noise-free setting to reduce external disturbances. The videos captured during the trials were subsequently analyzed with EthoVision XT software (Noldus, Information Technology, Wageningen, The Netherlands).(Torabi et al., 2020).

### 2.7 Perfusion and tissue preparation

In the eighth week of the study, the rats received deep anesthesia via an intraperitoneal injection of ketamine (80 mg/kg) and diazepam (10 mg/kg). After achieving anesthesia, cardiac perfusion was performed on the animals using 150 mL of normal saline, followed by 200 mL of paraformaldehyde (4 %). The spinal cords were then quickly removed and fixed in paraformaldehyde (4 %) at 4°C for a duration of 4–5 days. Afterward, conventional histological techniques were employed to process the spinal cord tissues, with samples embedded in paraffin blocks for subsequent sectioning. For histological analysis, tissue sections were stained using hematoxylin and eosin (H&E) according to standard protocols. The stained slides were subsequently analyzed and photographed using a microscope fitted with a camera. Image J software was used to evaluate the volume of the lesion cavity that formed in the spinal cord.

### 2.8 Stereological estimation

The first step in the tissue processing involved slicing the samples into serial sections, each measuring 5 µm in thickness. For the purpose of stereological analysis, 10 tissue sections were randomly selected. The total sections collected from each sample were divided by 10 to establish the sampling interval. The initial section was randomly selected from a range of 0–10, with subsequent sections chosen at this defined interval from the previous one. This method resulted in the random selection of 10 sections per sample. The tissue volume was determined using the Cavalieri technique, employing the following formula:

V = ∑p. a/p.t

### 2.9 Quantification of neurons and glial cells

The numbers of neurons and glial cells in the spinal cord were quantified using unbiased stereological methods. After tissue processing, serial 5-µm paraffin sections were prepared, and 10 sections per animal were selected using Systematic Uniform Random Sampling (SURS) to ensure representative and unbiased sampling.

Cell quantification was performed using the optical dissector method. Neurons and glial cells were identified based on morphological criteria under high magnification. Neuronal density (Nv) was calculated using the following formula:

Nv = (ΣQ^- / (h × a/f × Σp)) × (t / BA)

where:•ΣQ⁻ = number of counted neurons,•h = dissector height,•a/f = area of the counting frame,•Σp = number of points hitting the reference space,•t = total number of sections,•BA = spinal cord reference area.

Glial cell density was quantified using the same optical dissector approach and expressed as cells × 1000/µm³ . The representative images used for quantification are shown in Fig. 5, and all analyses were performed blinded to treatment groups to avoid bias (Afshar et al., 2021).

### 2.10 Western blotting

The samples were processed by homogenizing them in a lysis buffer containing 150 mM NaCl, 50 mM Tris-HCl (pH 7.4), Triton X-100, 10 % glycerol, 5 mM EDTA, 1 %, and protease inhibitors. After homogenization, the samples were heated and then centrifuged to obtain the supernatant. Protein concentrations were measured, and the samples were mixed with SDS loading buffer (5 ×). For protein separation, an SDS-PAGE gel (10 %) was utilized, followed by transferring the proteins to an Immobilon-PSQ PVDF membrane (Millipore Sigma). The membrane was then incubated overnight at 4°C with primary antibodies (IL-1β: Rabbit polyclonal antibody (Cell Signaling Technology, #12703; dilution 1:1000, IL-6: Mouse monoclonal antibody (Abcam, ab9324; dilution 1:1000, Caspase-3: Rabbit polyclonal antibody (Cell Signaling Technology, #9662; dilution 1:1000, β-actin: Mouse monoclonal antibody (Sigma-Aldrich, A5441; dilution 1:5000), used as a loading control. After incubation with primary antibodies, membranes were washed three times with TBST and incubated with appropriate HRP-conjugated secondary antibodies (goat anti-rabbit; Cell Signaling Technology) at a dilution of 1:5000 for 2 h at room temperature. Band intensities were quantified using Image J software, and target protein levels were normalized to β-actin (Khoshsirat et al., 2021).

### 2.11 Measurement of reduced glutathione (GSH) content, Glutathione disulfide (GSSG) and Reactive oxygen species (ROS)

To measure the levels of reduced glutathione (GSH) and glutathione disulfide (GSSG), a working solution was prepared with 50 μl of 5,5’-Dithiobis-(2-nitrobenzoic acid) (DTNB, Sigma-Aldrich), 100 μl of Tris (Sigma-Aldrich), and 840 μl of distilled water. This solution was quantified using a spectrophotometer. Next, 10 μl of tissue lysate was added to 990 μl of the DTNB mixture, mixed thoroughly, and allowed to incubate for 5 min at room temperature. The GSH concentration was measured in microliters. DTNB, also called Ellman’s Reagent, served to detect thiol groups, improving the sensitivity of total glutathione detection via a recycling reaction. The resulting yellow compound, 2-nitro-5-thiobenzoic acid, allowed for GSH quantification by measuring its absorbance at 412 NM. For ROS protocol Spinal cord tissues (50–100 mg) were homogenized in 10 volumes of PBS or RIPA buffer and centrifuged at 13,000 rpm for 10 min at 4°C. Supernatants were collected for ROS analysis. A 10 mM DCF-DA stock solution was prepared in DMSO and diluted in PBS to a working concentration of 10–20 µM. Tissue lysates (100 µL) were incubated with 100 µL of DCF-DA solution at 37°C in the dark for 30–60 min. Absorbance was measured at 500–530 nm using a microplate reader. ROS levels were normalized to protein content determined via BCA assay and expressed as absorbance per mg protein. Blank (PBS + DCF-DA), negative (lysate without DCF-DA), and positive controls (e.g., H₂O₂-treated lysates) were included. All samples were run in triplicate.

### 2.12 Real-time PCR

From the cellular samples, RNA was isolated, and its quality and purity were assessed. Following this evaluation, reverse transcription was conducted in accordance with the protocols outlined in the cDNA synthesis kit. The expression levels of the target miRNAs (miR-19a-3p, miR-19b-3p, miR-24, miR-27b) will subsequently be analyzed using the SYBR Green Real-Time PCR technique (Table 1). Relative gene expression was calculated using the 2^-ΔΔCt^ method, with GAPDH as the internal control (Darabi et al., 2017, Nooraei et al., 2018).

**Table 1:** Primer sequence.

Gene	Primer sequence	Size (bp)	Temperature
miR-19a-3p	Forward: 5′- TGTGCAAATCTATGCAAAAC −3′ Reverse: 3′- GTGCAGGGTCCGAGGTATT −5′	185	56
miR-19b-3p	Forward:5′- TGTGCAAATCCATGCAAAAC −3′ Reverse:3′- GTGCAGGGTCCGAGGTATT −5′	212	58
miR-24	Forward:5'TGGCTCAGTTCAGCAGGAACA −3′ Reverse: 3′- GTGCAGGGTCCGAGGTATT −5′	194	62
miR-27b	Forward: 5′- TTCACAGTGGCTAAGTTCCG −3′ Reverse: 3′- GTGCAGGGTCCGAGGTATT −5′	190	58
GAPDH	F: 5′-ACTGTCTCCTGCGACGCTA-3′ R: 3′-CGTGGTCCAGGGTTTCTAT-5′	188	54

### 2.13 Immunohistochemistry

Immunohistochemistry was performed on transverse spinal cord cryosections (20 µm) to evaluate neuronal survival (NeuN) and astrocytic reactivity (GFAP). Sections were rinsed in PBS and incubated in blocking solution containing 5 % normal donkey serum (Sigma-Aldrich, St. Louis, MO, USA) and 0.3 % Triton X-100 for 1 h at room temperature. For neuronal labeling, sections were incubated overnight at 4 °C with mouse anti-NeuN primary antibody (1:500; Cat# MAB377, Millipore, Burlington, MA, USA), followed by donkey anti-mouse IgG Alexa Fluor 488 secondary antibody (1:1000; Cat# A-21202, Thermo Fisher Scientific, Waltham, MA, USA). For astrocytic labeling, adjacent serial sections were incubated with rabbit anti-GFAP primary antibody (1:500; Cat# ab7260, Abcam, Cambridge, UK), followed by donkey anti-rabbit IgG Alexa Fluor 488 secondary antibody (1:1000; Cat# A-21206, Thermo Fisher Scientific). All sections were counterstained with DAPI (1 µg/mL; Cat# D9542, Sigma-Aldrich) and mounted with Fluoromount-G (Cat# 0100–01, SouthernBiotech, Birmingham, AL, USA). Images were acquired using a fluorescence microscope (Carl Zeiss, Germany) (Abbaszadeh et al., 2014, Darabi et al., 2013).

### 2.14 Statistical analysis

Statistical analyses were performed using SPSS version 18. Quantitative data were presented as mean ± standard deviation (SD). The Kolmogorov-Smirnov test was utilized to assess the normality of the data distribution. For datasets meeting the criteria for normality, one-way ANOVA was conducted, followed by Tukey's post hoc analysis. Conversely, for datasets that did not adhere to normal distribution, the non-parametric Kruskal-Wallis test and Dunn's post hoc analysis were employed. A significance threshold of P < 0.05 was considered statistically significant.

## 3 Result

### 3.1 Characterization of exosome derived from HUCB

**Fig. 1:**
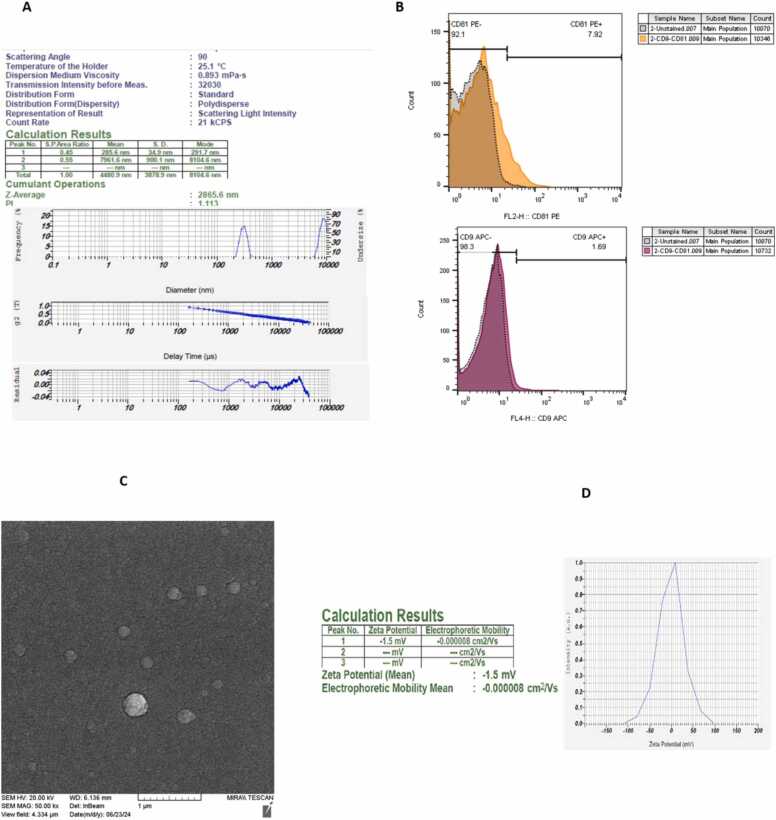
Characterization of exosomes derived from human umbilical cord blood (HUCB-exosomes) included the following findings: (A) Dynamic light scattering (DLS) analysis revealed a consistent distribution of particle sizes. (B) Flowcytometry confirmed the presence of specific exosome markers, CD9 and CD81. (C) Scanning electron microscopy (SEM) imaging revealed that HUCB-exosomes were primarily spherical vesicles. (D) Zeta potential analysis assessed the surface charge of the exosomes, indicating their stability while in suspension.

HUCB-derived exosomes were characterized through several techniques, including scanning electron microscopy (SEM), flow cytometry, Zeta potential analysis and dynamic light scattering (DLS). SEM imaging revealed that the HUCB-derived exosomes were mainly spherical in shape (Figs. 1A, 1B, 1C, 1D). DLS results demonstrated a consistent particle size distribution, with diameters falling within the range of 100–200 nm. Flowcytometry provided additional validation of the exosomes' identity by confirming the presence of specific markers, CD9 and CD81.

### 3.2 Functional and behavior result

**Fig. 2:**
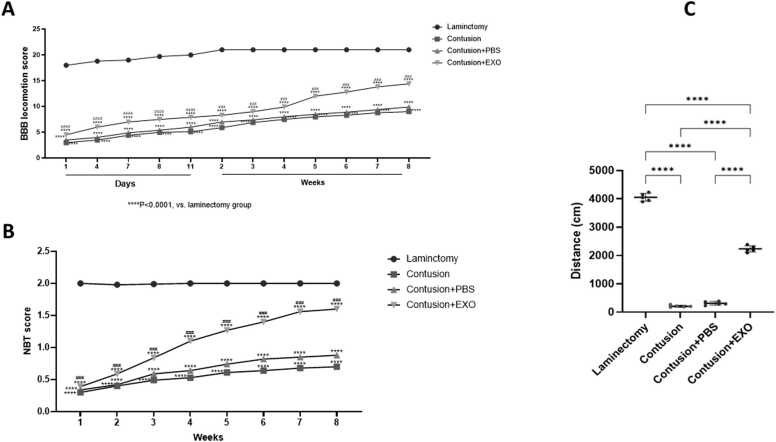
(A) Recovery of motor function over an eight-week period following SCI, as assessed by the BBB test. The graph displays the BBB scores from day 1 through week 8 for the four experimental groups. Notably, animals in the exosome-treated group exhibited significantly enhanced functional recovery in comparison with those in the contusion group (**** P < 0.0001). (B) Sensory-motor coordination was evaluated using the Narrow Beam Test (NBT) over an eight-week period following SCI. The performance of the four experimental groups is illustrated in the graph. Prior to the SCI induction, all rats navigated the beam without difficulty. However, post-injury, they displayed significant challenges in traversing the beam, exhibiting poor foot placement. By the conclusion of the experiment, the group treated with exosomes demonstrated substantial improvement in performance compared to the contusion group, with a significance level of **** P < 0.0001. (C) Assessment of locomotor activity using the open-field test among the experimental groups. The graph illustrates the distances traveled by the rats throughout the experiment. Rats in the exosome treatment group demonstrated significantly higher locomotor activity, traveling greater distances compared to those in the contusion-only group. In contrast, the contusion group exhibited notably lower levels of locomotor activity(**** P < 0.0001).

The evaluation of motor function was conducted using the BBB motor rating scale over an eight-week period following the establishment of the SCI contusion model in all experimental groups. Weekly assessments demonstrated a gradual improvement in BBB scores beginning immediately after the injury. Significantly, the animals treated with exosomes showed a much greater recovery of function compared to those in the contusion group (Fig. 2A). Prior to the induction of SCI, the animals were able to cross the narrow beam effortlessly, showing no difficulties with foot placement. However, following SCI induction, they faced considerable challenges in navigating the beam and exhibited a marked decline in proficiency. Statistical analysis revealed a significant enhancement in performance for the contusion+ exosome group compared to the contusion group (Fig. 2B). The open-field test was performed to assess the overall levels of locomotor activity. Rats treated with exosomes traveled significantly greater distances compared to those in the contusion and PBS-treated groups. In contrast, the contusion and PBS groups exhibited markedly reduced locomotor activity levels, with a significance level of (Fig. 2C).

### 3.3 IL-1β, IL-6, and caspase-3 expression

**Fig. 3:**
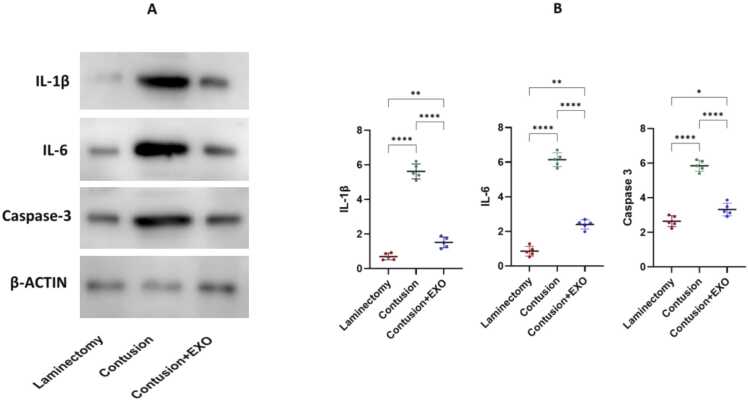
A Western blot analysis was conducted eight weeks post-SCI to assess the changes in levels of IL-1β, IL-6, Caspase-3, and β-actin (A)**.** The results revealed a notable reduction in the expression of these markers in the exosome-treated group when compared to the contusion groupQuantification represents the mean of three biological replicates (* P < 0.05, ** P < 0.01, **** P < 0.0001) (B).

We conducted Western blot analysis eight weeks post-SCI to assess changes in IL-1β, IL-6, and Caspase-3 level. The analysis revealed a significant increase in these markers in the contusion group (Fig. 3). In contrast, the exosome-treated group exhibited reduced levels of these proteins in comparison with the contusion group. To confirm that these findings were not batch-dependent, we independently repeated the experiment using a newly prepared exosome batch, and the results showed the same trend. These results indicate that exosome therapy may have anti-inflammatory effects following SCI.

### 3.4 Cavity formation and spinal cord volume

**Fig. 4:**
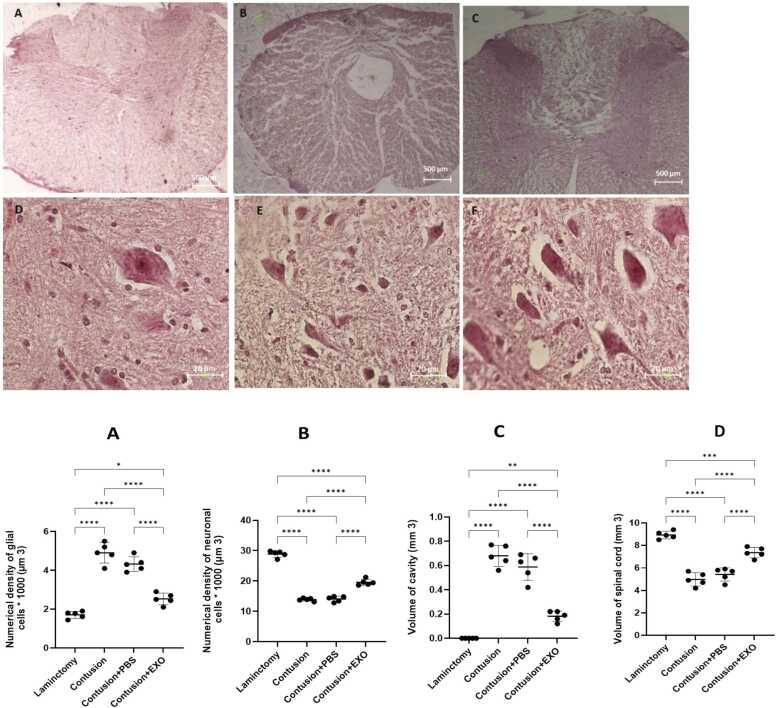
Sections of the spinal cord from the four experimental groups were stained with hematoxylin-eosin (H&E): A, D) Laminectomy only; B, E) Contusion injury; C, F) Contusion + Exosomes. The stained sections were photographed using a microscope equipped with an integrated camera (Upper panel A-F). In the lower panel the assessment of Neural Cell Density, spinal cord and cavity volume (mm³). Representative images used for the statistical quantification shown in panels A–D are provided in Fig. 4. (A) Glial Cell Density: The analysis of glial cell density (cells ×1000/µm³) from the representative images demonstrated that the exosome-treated group exhibited lower glial cell density, reflecting a reduction in gliosis compared to the other groups. (B) Neural Cell Density: The quantification of neural cell density (cells × 1000/µm³) quantified from the representative images, indicated that the exosome-treated group had a higher neural cell density relative to the contusion-only and PBS-treated groups. (C) Cavity volume demonstrated a marked reduction in the exosome-treated group when compared to both the contusion and PBS-treated groups, with no cavity observed in the laminectomy group. (D) Spinal volume demonstrated a marked increase in the exosome-treated group compared to both the contusion and PBS-treated groups (* P < 0.5, ** P < 0.01, *** P < 0.001, **** P < 0.0001) receptively.

Histological analyses conducted eight weeks following the injury demonstrated a significant reduction in cavity volume in the exosome-treated group compared to the contusion groups. Notably, there was no formation of a central cavity observed in the laminectomy group (Fig. 4. Upper panel). In lower panel, the quantification of neural cell density in spinal cord sections across the four experimental groups is depicted. The exosome-treated group displayed a higher density of neural cells than the contusion groups (Fig. 4). Furthermore, assessments of glial cell density in spinal cord sections indicated that the exosome-treated group had lower glial cell density, suggesting a reduction in gliosis relative to the other groups.

### 3.5 ROS, GSSG, GSH analysis

**Fig. 5:**
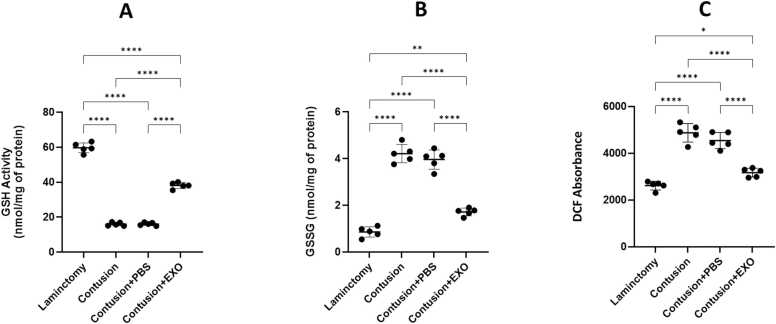
Comparative analysis of oxidative stress markers across the four experimental groups: Laminectomy, Contusion, Contusion + PBS, and Contusion + Exosomes. The figure is divided into three sections: (A) The graph displays GSH (glutathione) activity quantified in nmol/mg of protein. The results indicate that both the Contusion and Contusion + PBS groups demonstrate reduced GSH activity compared to the Laminectomy group. In contrast, the Contusion + Exosomes group exhibits a notable restoration of GSH levels, highlighting the beneficial effect of exosome treatment. (B) GSSG Levels: The graph depicts GSSG (oxidized glutathione) levels, also measured in nmol/mg of protein, across the experimental groups. The pattern mirrors that of section A, with elevated GSSG levels in the Contusion and Contusion + PBS groups, and a significant decrease in the Contusion + Exosomes group, reinforcing the exosomes' role in alleviating oxidative stress post-SCI. (C) DCF Absorbance: A significant increase in DCF absorbance is observed in the Contusion and Contusion + PBS groups, indicating heightened oxidative stress. In contrast, the Contusion + Exosomes group demonstrates a notable reduction in absorbance, suggesting that exosome treatment may mitigate SCI-induced oxidative stress (**P < 0.001, **** P < 0.0001).

The examination of oxidative stress markers across the experimental groups reveals notable results, as depicted in Fig. 5. DCF absorbance, which serves as an indicator of reactive oxygen species (ROS) levels, is significantly higher in the Contusion and Contusion + PBS groups, indicating increased oxidative stress post SCI. In contrast, the Contusion + Exosomes group shows a marked decrease in DCF absorbance, nearly returning to the levels seen in the Laminectomy control group, suggesting that exosome treatment effectively reduces ROS production. Additionally, GSH activity, an important measure of antioxidant capacity, is significantly lower in the Contusion and Contusion+PBS groups in comparison with the Laminectomy group (Fig. 5). Conversely, exosome treatment restores GSH activity to levels close to the control group, demonstrating the potential of exosomes to protect antioxidant defenses after SCI. Finally, GSSG levels exhibit a similar trend to DCF absorbance, with elevated levels in the Contusion and Contusion + PBS groups and a significant reduction in the Contusion + Exosomes group, further reinforcing the role of exosomes in reducing oxidative stress and promoting recovery post-SCI.

### 3.6 gene expression result

**Fig. 6:**
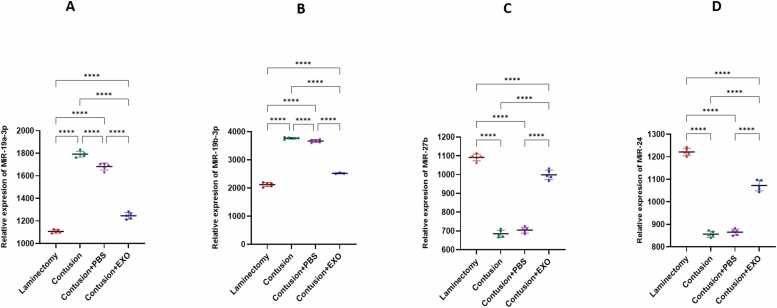
Real time PCR analysis of miR-19a-3p, miR-19b-3p, miR-27b and miR-24 expression across the experimental groups: Laminectomy, Contusion, Contusion + PBS and Contusion + Exosomes. The findings reveal that exosome therapy leads to a notable reduction in miR-19a-3p (A) and miR-19b-3p (B) expression, suggesting that the exosome-treated group exhibits significantly lower levels of miR-19a-3p and miR-19b-3p compared to the Contusion group and notable increase in miR-27b (C) and miR-24 (D) in the exosome-treated group in comparison with contusion group (**** P < 0.0001).

The results of the Real time PCR analysis of miR-19a-3p, miR-19b-3p, miR-27b and miR-24 indicate significant changes in gene expression across the experimental groups. The expression level of miR-19a-3p and miR-19b-3p is significantly elevated in the Contusion group compared to the Contusion+ Exosomes group. In contrast, the Contusion+ Exosomes group displays a markedly increase expression level of miR-27b and miR-24 relative to the Contusion group. Overall, exosome therapy is associated with a reduction in the expression of both miR-19a-3p and miR-19b-3p gene, highlighting its potential protective effects following SCI (Fig. 6).

### 3.7 Immunohistochemical staining for Neu-N and GFAP revealed marked differences among the experimental groups

**Fig. 7:**
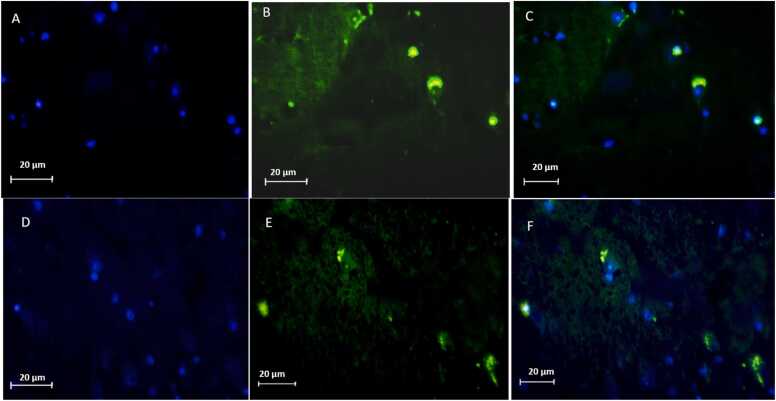
Representative immunofluorescence images of spinal cord sections from two experimental groups. Contusion (A, B, C), and Contusion + Exosome (D, E, F). DAPI counterstain (A, D) with NeuN staining (B, E) and merge (C, F), showing neuronal distribution. In the contusion group, NeuN signal is markedly reduced, In the exosome-treated group, NeuN signal is partially preserved compared with the contusion group.

**Fig. 8:**
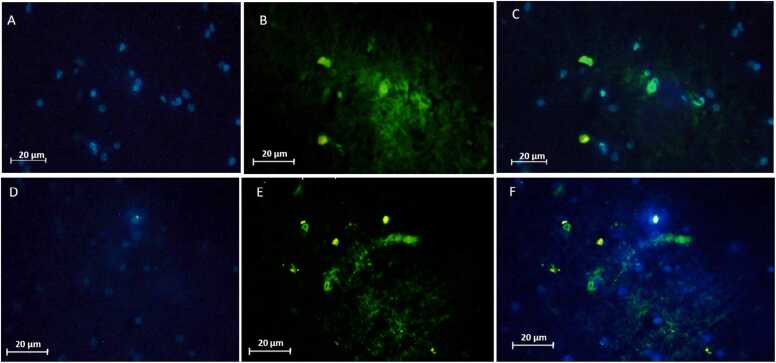
Representative immunofluorescence images of spinal cord sections from two experimental groups. Contusion (A, B, C), and Contusion + Exosome (D, E, F). DAPI counterstain (A, D) with GFAP staining (B, E) and merge (C, F). In the contusion group, GFAP expression is upregulated with hypertrophic astrocytes forming dense glial scarring. In the exosome-treated group GFAP reactivity is attenuated compared with the contusion group.

In the contusion injury group (A, B, C,upper panel), a pronounced reduction in NeuN-positive neurons was observed at the lesion epicenter, indicating substantial neuronal loss compared with the contusion+ EXO group. In the contusion + exosome treatment group (E, F, G) NeuN staining demonstrated partial preservation of neuronal density and morphology within and around the lesion area compared to the untreated contusion group. In parallel, GFAP immunoreactivity was significantly upregulated in the contusion injury group (A, B, C) in the lower panel, with astrocytes forming dense glial scars surrounding the injury site. These findings reflect both neuronal degeneration and robust reactive astrogliosis after traumatic SCI. GFAP expression was attenuated, showing reduced astrocytic hypertrophy and less extensive glial scar formation. Together, these results suggest that exosome treatment (E, F, G) mitigated neuronal loss while modulating astrocytic reactivity in the injured spinal cord (Fig. 7, Fig. 8).

## 4 Discussion

The present study investigates the therapeutic potential of exosomes derived from hUCB in the treatment of SCI, focusing on their effects on various molecular and cellular markers associated with injury and recovery. Our findings underscore the promising role of exosome therapy in modulating key pathological processes following SCI, particularly through miRNA regulation, neuroprotection, and inflammation suppression. Numerous studies support the therapeutic potential of exosomes in SCI, demonstrating their ability to reduce inflammation, apoptosis, and oxidative stress, while enhancing neurogenesis and functional recovery (Hu et al., 2022, Luan et al., 2024). Our findings in behavioral assessments, including BBB, NBT and open-field test demonstrate that hUCB-Exosome ability to improve motor function in SCI, consistent with prior investigations on small extracellular vesicles (sEVs) and mesenchymal stem cell (MSC)-derived exosomes. Although the open-field test is commonly used to evaluate locomotor recovery after SCI, it is also sensitive to emotional factors such as anxiety and depression, which are common following SCI. In our rat contusion model treated with intravenous exosomes, changes in movement patterns may reflect not only motor improvements but also behavioral or affective alterations. Therefore, results from this test should be interpreted with caution, and future studies should incorporate complementary assessments to distinguish motor from emotional outcomes. A growing body of evidence points to the anti-apoptotic, anti-inflammatory, and neuroprotective properties of exosome derived from hUC-MSC in various models of neural injury (Kang & Guo, 2022). Kang and Guo (2022) reported that exosome derived from hUC-MSC facilitates functional recovery in a rat model of SCI. This recovery is achieved by inhibiting apoptosis and decreasing glial activation via the Wnt/β-catenin signaling pathway. This finding aligns with our results, which demonstrate that hUCB-Exos reducing pro-apoptotic markers and enhancing neuroprotection (Kang & Guo, 2022). Similarly, Zhong-Xia Zhang et al. (2023) demonstrated that exosome derived from hUC-MSC reduce inflammation mediated by microglia in a Parkinson's disease (PD) model, providing neuroprotection by inhibiting pyroptosis and inflammation. These results closely resemble our findings regarding the anti-inflammatory effects and glial inhibition observed in SCI models (Z.-X. Zhang et al., 2023). Western blot analyses in this study revealed a notable reduction in IL-1β, IL-6, and Caspase-3 level in the exosome-treated group compared to the contusion controls. IL-1β is a marker that reflects microglial activation (Gąssowska-Dobrowolska et al., 2023, Lafrenaye et al., 2020). The reduction in these markers suggests that exosome therapy effectively attenuates microglial activation, which are critical contributors to secondary damage and impaired recovery post-SCI. The suppression of glial scar formation is particularly noteworthy, as it may facilitate axonal regeneration and functional recovery. This decrease implies that exosome therapy not only fosters neuroprotection but also possesses anti-inflammatory properties, reinforcing its potential as an effective therapeutic approach for managing spinal cord injuries (Poongodi et al., 2024). Furthermore, a related study showed that exosomes derived from human umbilical cord mesenchymal stem cells (hUC-MSC) significantly enhanced motor function in a rat model of SCI. This research revealed that exosome treatment led to decreased transcription levels of the IL-1β, IL-6 signifying a reduction in the inflammatory response linked to SCI. Additionally, the anti-apoptotic effects of hUC-MSC-derived exosomes, reduced levels of and Caspase-3, further emphasize the therapeutic potential of these exosomes in promoting recovery from spinal cord injuries (Kang & Guo, 2022). Recent research by Zhiwei Luan et al. (2024) further emphasizes the anti-inflammatory effects of mesenchymal stem cell (MSC)-derived exosomes, demonstrating their ability to inhibit the NF-κB/MAPK signaling pathway in the context of SCI, leading to reduced production of pro-inflammatory cytokines and ROS. This finding aligns with our results on hUCB-derived exosomes, which effectively downregulate pro-inflammatory cytokines like IL-1β and IL-6, highlighting their anti-inflammatory and antioxidant roles in the context of SCI (Luan et al., 2024). Adding to this body of evidence, the work by Xinyuan Hu et al. (2022) introduces sEVs derived from hUC-MSCs as a novel therapeutic strategy for SCI. Histopathological analysis indicated that the size of the cavity formed in the spinal cord was reduced in the exosome-treated group when compared to the contusion group. This suggests that exosome therapy alleviates tissue damage. In a related study, exosomes derived from neural stem cells (NSCs-Exos) significantly enhanced microvascular regeneration, minimized spinal cord cavity size, and facilitated improved recovery of motor function in rat with spinal cord injury. These results further support the therapeutic benefits of exosome treatment in fostering tissue repair and functional recovery after SCI (Zhong et al., 2020). Furthermore, cell counting revealed a decreased number of glial cells alongside an increased number of neural cells in the exosome treatment group. This alteration in cell populations indicates that exosome therapy not only diminishes gliosis but also fosters neurogenesis and enhances neuronal survival, leading to better structural and functional results. In a similar investigation, exosomes isolated from olfactory ensheathing cells (OECs) demonstrated neuroprotective properties in SCI by influencing microglial phenotypes and supporting neuronal survival. The OEC-derived exosomes were shown to suppress the polarization of pro-inflammatory macrophages and microglia while enhancing the prevalence of anti-inflammatory cells. This observation aligns with our findings regarding the capacity of exosome therapy to alter cellular dynamics, promoting a more neuroprotective environment (Fan et al., 2022). Our examination of oxidative stress markers revealed that levels of ROS and GSSG were elevated in the contusion control group, whereas GSH levels were significantly increased in the exosome-treated group. This suggests that exosome therapy bolsters antioxidant defenses and mitigates oxidative stress, a key contributor to SCI-induced damage. By scavenging ROS and enhancing cellular antioxidant mechanisms, exosomes foster a more conducive environment for tissue repair and regeneration. Their study revealed that intra-lesion administration of hUC-MSC-sEVs led to a marked enhancement in motor function in in SCI rats, as indicated by higher BBB scores (Hu et al., 2022). Notably, hUC-MSC-derived sEVs have been shown to enhance neurogenesis and diminish inflammation, aligning with our findings regarding the neuroprotective and anti-inflammatory properties of hUCB-derived exosomes. Additionally, Hu et al. identified the ERK1/2 signaling pathway as a crucial mediator in the activation of NSCs, facilitating their proliferation and differentiation. This pathway presents a novel mechanism by which hUC-MSC-sEVs contribute to tissue repair and recovery following spinal cord injury. While the activation of NSCs by sEVs differs from the modulation of glial cells by hUCB-Exos, it introduces an additional dimension to the potential cellular targets and therapeutic effects of mesenchymal stem cell-derived exosomes and sEVs in the treatment of spinal cord injuries. In summary, these studies highlight the common mechanisms by which exosomes and sEVs derived from human umbilical cord mesenchymal stem cells contribute to recovery from SCI. They operate through various pathways, including the inhibition of inflammation via the NF-κB/MAPK signaling pathway(Luan et al., 2024), modulation of the Wnt/β-catenin pathway to reduce apoptosis and glial activation (Kang & Guo, 2022), and activation of NSCs through the ERK1/2 pathway to enhance neurogenesis (Hu et al., 2022). Other aspects of this study was its focus on miRNAs which are critical post-transcriptional regulators that influence inflammation, apoptosis, and neurogenesis following SCI. Our real-time PCR analysis revealed that miR-19a-3p and miR-19b-3p were significantly upregulated in the contusion group but were downregulated following exosome therapy, whereas miR-27b and miR-24 were significantly increased in the exosome-treated group. miR-19a-3p and miR-19b-3p are members of the miR-17–92 cluster and have been shown to promote pro-inflammatory signaling, apoptosis, and secondary injury in various CNS disorders. Their upregulation in the contusion group is consistent with previous studies linking these miRNAs to enhanced neuroinflammation and neuronal damage (Acharya et al., 2020, Silvestro and Mazzon, 2022, Tregub et al., 2023). However, their downregulation following exosome therapy suggests that hUCB-derived exosomes may exert neuroprotective effects by suppressing miRNA-mediated inflammatory cascades. miR-27b and miR-24, on the other hand, are associated with anti-inflammatory and neuroprotective effects (Gaudet et al., 2018). Studies have shown that miR-27b plays a key role in suppressing microglial activation and reducing IL-6 and TNF-α expression, thereby shifting the inflammatory response toward a more neuroprotective state (Li et al., 2021). Similarly, miR-24 has been implicated in neuronal survival and axonal regeneration following CNS injury (Liu et al., 2022, Pan et al., 2021). The upregulation of these miRNAs in the exosome-treated group suggests that hUCB-derived exosomes may facilitate functional recovery by enhancing neuroprotective and anti-inflammatory miRNA expressions. These findings provide further evidence that exosome therapy not only modulates protein expression but also fine-tunes post-transcriptional gene regulation through miRNA delivery, underscoring a novel mechanism by which exosomes exert their neuroprotective effects in SCI.

## 5 Conclusion

hUCB-derived exosomes significantly improve functional and molecular outcomes following SCI. By modulating miRNA expression, reducing glial activation, enhancing neuronal survival, suppressing inflammation, and mitigating oxidative stress, exosome therapy presents a powerful and multifaceted strategy for SCI treatment. Exosome therapy not only exerts anti-inflammatory and neuroprotective effects but also plays a crucial role in post-transcriptional gene regulation. These findings open new avenues for the development of miRNA-based exosome therapies for SCI and other neurodegenerative disorders.

## CRediT authorship contribution statement

**Abbaszadeh Hojjat Allah:** Writing – original draft, Supervision. **Hamid Reza Mosleh:** Investigation, Formal analysis. **Shahram Darabi:** Formal analysis, Data curation. **Foozhan Tahmasebinia:** Investigation. **Amirreza Beirami:** Formal analysis. **Shima Jahanbaz:** Formal analysis, Data curation. **Hooman Kazemi Mirni:** Methodology. **Maryam Bahrami:** Methodology, Investigation. **Leila Darabi:** Methodology, Investigation. **Hadise Taheri:** Methodology, Investigation. **Reza Bahar:** Software, Resources. **Maral Hasanzadeh:** Investigation. **Abbas Aliaghaei:** Investigation. **Amin Karamian:** Methodology, Investigation.

## Ethical statement

This study was conducted with the approval and support of Shahid Beheshti University Medical Research Ethics Committee (IR.SBMU.LASER.REC.1404.033). All animal experiments were performed in compliance with the ARRIVE guidelines and in accordance with the U.K. Animals (Scientific Procedures) Act, 1986, EU Directive 2010/63/EU, and the National Institutes of Health guide for the care and use of laboratory animals (NIH Publications No. 8023, revised 1978). All necessary efforts were made to minimize animal suffering.

## Funding

This study received financial support from the Laser Application in Medical Sciences Research Center, Shahid Beheshti University of Medical Sciences, Tehran, Iran.

## Declaration of Competing Interest

The authors declare that there is no conflict of interest.
